# Comparative *in vitro* evaluation of CAD/CAM vs conventional provisional crowns

**DOI:** 10.1590/1678-775720150451

**Published:** 2016

**Authors:** Adil Othman ABDULLAH, Effrosyni A TSITROU, Sarah POLLINGTON

**Affiliations:** 1 - Erbil Polytechnic University, Department of Dental Prevention, Erbil, Kurdistan Region, Iraq.; 2 - Aristotle University of Thessaloniki, Department of Operative Dentistry, School of Dentistry, Thessaloniki, Greece.; 3 - University of Sheffield, School of Clinical Dentistry, Academic Unit of Restorative Dentistry, Sheffield, United Kingdom.

**Keywords:** Temporary dental restoration, Dental marginal adaptation, CAD-CAM, Dental impression technique, Dental crowns, Dental prosthesis

## Abstract

**Objective:**

This study compared the marginal gap, internal fit, fracture strength, and mode of fracture of CAD/CAM provisional crowns with that of direct provisional crowns.

**Material and Methods:**

An upper right first premolar phantom tooth was prepared for full ceramic crown following tooth preparation guidelines. The materials tested were: VITA CAD-Temp^®^, Polyetheretherketone “PEEK”, Telio CAD-Temp, and Protemp™4 (control group). The crowns were divided into four groups (n=10), Group1: VITA CAD-Temp^®^, Group 2: PEEK, Group 3: Telio CAD-Temp, and Group 4: Protemp™4. Each crown was investigated for marginal and internal fit, fracture strength, and mode of fracture. Statistical analysis was performed using GraphPad Prism software version 6.0.

**Results:**

The average marginal gap was: VITA CAD-Temp^®^ 60.61 (±9.99) µm, PEEK 46.75 (±8.26) µm, Telio CAD-Temp 56.10 (±5.65) µm, and Protemp™4 193.07(±35.96) µm (P<0.001). The average internal fit was: VITA CAD-Temp^®^ 124.94 (±22.96) µm, PEEK 113.14 (±23.55) µm, Telio CAD-Temp 110.95 (±11.64) µm, and Protemp™4 143.48(±26.74) µm. The average fracture strength was: VITA CAD-Temp^®^ 361.01 (±21.61) N, PEEK 802.23 (±111.29) N, Telio CAD-Temp 719.24 (±95.17) N, and Protemp™4 416.40 (±69.14) N. One-way ANOVA test showed a statistically significant difference for marginal gap, internal gap, and fracture strength between all groups (p<0.001). However, the mode of fracture showed no differences between the groups (p>0.05).

**Conclusions:**

CAD/CAM fabricated provisional crowns demonstrated superior fit and better strength than direct provisional crowns.

## INTRODUCTION

Provisional restorations are used as an intermediate stage for short or long-term placement on teeth between the time of tooth preparation until the definitive indirect restorations are fabricated and placed^4^ ^,^ ^23^. The demand for tooth-colored restorations has significantly increased in recent years because of improved techniques and patient’s demand. Therefore, using various new restorative materials, which have excellent mechanical properties, are essential for both provisional and definitive restorations^1^ ^,^ ^9^.

The emergence of computer-aided design/computer-aided manufacture (CAD/CAM) technology in dentistry has allowed the successful use of different materials^7^. Using these systems to fabricate fixed restorations has gained popularity in comparison with conventional techniques^1^. In addition, this technology permits shaping of materials with high precision that cannot be easily carried out via a traditional method to make a dental restoration, and this technology now includes the fabrication of provisional restorations^13^.

Accurate provisional restorations are essential and serve a number of functions including protection of the pulpal tissues, bacterial contamination, and preservation of periodontal tissues. In addition, preventing rotation of the tooth from its normal position in terms of supra or infra occlusion, maintaining esthetics and oral functions, such as mastication and speech, is paramount^1^ ^,^ ^16^.

There are two major approaches for fabrication of provisional crowns: direct and indirect methods. A variety of new provisional blocks are available to use with the CEREC3 CAD/CAM system (Sirona, Bensheim, Germany). These materials can withstand the milling process because of their high strength^7^. However, conventional provisional materials cannot be prepared via the milling process, but require to be fabricated manually. There are certain difficulties associated with this manual technique, for example, inadequate surface texture and insufficient mechanical properties such as flexural strength^1^. It has been reported that CAD/CAM provisional crowns were stronger and exhibited better marginal accuracy than directly fabricated bis-acryl composite crowns, especially following thermal cycling. Therefore, using CAD/CAM may resolve these issues^26^.

The aim of this study was to compare the marginal and internal fit, fracture strength, and mode of fracture of provisional CAD/CAM crowns compared with chair side directly made provisional crowns. The null hypothesis tested was twofold: the CAD/CAM provisional crowns will have a better marginal and internal fit than direct counterparts. In addition, the CAD/CAM provisional crowns will provide superior strength to that of the directly fabricated crowns.

## MATERIAL AND METHODS

An upper right first premolar phantom tooth (Frasaco A3, Frasaco Franz Sachs & Co., GmbH, Germany) was prepared for a full ceramic crown with 1.5 mm occlusal reduction, the convergence angle of the wall was prepared to be approximately 6° and a round shoulder of 1 mm using a high speed hand piece operating with water coolant. A paralleling device (Nesor product LTD, Britain) was used during the preparation to enhance reproducibility of the preparation. An impression of the preparation was fabricated with vinyl-polysiloxane (Dublisil 15, Dreve Dentamid GmbH, Germany) to fabricate a master die from polyurethane base resin (AlphaDie^TM^MF, Schütz Labortechnik, Germany).

The CEREC InEos system (Sirona, Bensheim, Germany) was used for scanning the preparation. The preparation was powdered with scan spray (Vita Zahnfabrik, Germany) using a propellant to provide a thin, even layer of powder. The preparation was then scanned and the crown designed using the CEREC 3D v3.60 software. The three CAD/CAM provisional blocks were used: Acrylate polymer material VITA CAD-Temp^®^ (VITA Zahnfabrik, Germany), Polyethertherketone PEEK (Invibio Biomaterial Company, UK), and Polymethyl methacrylate material Telio CAD-Temp (Ivoclar Vivadent, Liechtenstein, Germany). Ten provisional crowns were milled for each group. The correlation mode was used with the spacer set at 10 microns for the three CAD/CAM groups and the default milling burs (1.2 mm cylinder bur, step bur) were used for the milling of the crowns. Following milling, the restorations were examined for the presence of any defects or cracks.

A fourth group consisted of ten provisional crowns made from Protemp^TM^4 (3M ESPE, Germany), which comprised the direct fabrication technique. An impression of the upper right first premolar phantom tooth was initially taken before any preparations were made to serve as an index impression for the fabrication of the provisional crowns. The materials used were: heavy bodied materials (Aquasil putty, blue and orange colour, Dentsply-Detrey GmbH, Germany) and light bodied material (Aquasil LV^TM^, green colour, Dentsply-Detrey GmbH, Germany); the manufacturer’s instructions were followed. The components of Protemp^TM^4 restorative material were mixed through a self-mixing gun and injected into the indexed impression. The indexed impression was placed on the master die until the mixed material completely set. Thus, ten direct provisional crowns were made. The crowns were finished and polished using rotary rubber cups (Sof-Lex™ Disc 3M ESPE, Germany) and were examined to detect any defects circumferentially.

### Measurement of marginal fit

The marginal gap was evaluated by means of a replica technique and a luting agent. A light-bodied silicone rubber impression material (Aquasil LV^TM^, green colour, Dentsply-Detrey GmbH, Germany) was used for the purpose of cementation. Each crown was filled with the light-bodied material and placed on the corresponding replica with a constant force of 40 N for 3 minutes using a universal tensometer (Lloyd Universal Testing Machine, LRX 2K5, Hants, UK). After setting of the silicone material, the crowns were carefully removed from the master die. A thin film of the light bodied impression material was adhered to the inner surface of the crown in all cases. In order to support the thin film, a heavy-bodied material of contrasting colours (Aquasil putty, blue and orange colour, Dentsply-Detrey GmbH, Germany) was used. After setting, an index was marked on a heavy-bodied material to provide a consistent series of locations (buccal, lingual, mesial, and distal) for sectioning, and each silicone replica was sectioned into smaller segments for microscopic examination. The marginal gap and internal fit were measured at nine different points both buccolingually and mesiodistally using the AxioVision Rel Microsoft Software (Carl Zeiss Microscopy version 4.7, Germany) with 10X magnification.

### Measurement of fracture strength

The crowns were cemented to the master die using TempBond NE (Kerr, CA, USA) with a standardized time of six minutes to allow for complete setting of the cement. The cement was mixed on a paper pad, following the manufacturer’s instructions. The crowns were filled with cement and seated on its corresponding master die. The cemented crowns were subjected to a static loading test after storage in water for 24 hours, without any thermal or load cycling fatigue.

Samples were loaded under a standard compression load at a crosshead speed of 1mm/minute and the force recorded using the universal testing machine (Lloyd Universal Testing Machine, LRX 2K5, Hants, UK) with a 2500 Newton loaded cell for three minutes. A plunger with a steel ball (4.24 mm diameter) was used to transmit the compressive force until fracture occurred. The ball was positioned in the middle of the occlusal plane, between the buccal and palatal cusps. A piece of rubber dam was placed as a stress breaker between each crown sample and the steel ball in order to remove any potential stress concentration during applying the load. Loading was continued until fracture occurred and fracture loads were recorded. After a first loading test, the master die, which was fabricated from AlphaDie and represented the abutment, was microscopically examined (Wild M3Z, Heerbrugg, Switzerland) to detect any deformation or cracks from occlusal surface and/or finishing line circumferentially.

The mode of fracture detected was also recorded for all samples. This was recorded depending on Burke’s classification, which comprises the following categories according to the pattern of crown fracture^3^.

Class I – Minimal fracture or crack in crown

Class II – Less than half of crown lost

Class III – Crown fracture through midline; half of crown displaced or lost

Class IV – More than half of crown lost

Class V – Severe fracture of tooth and/or crown

### Statistical analysis

All data about the different fabrication technique and materials used were analyzed. The average of marginal gap, internal fit, fracture strength, and standard deviations were calculated using one-way analysis of variance (ANOVA) followed by *post hoc* tests (Tukey’s test). However, the data of mode of fracture were compared using Kruskal-Wallis non-parametric test. The p-value less than 0.05 (p<0.05) was considered as statistically significant. All the graph, calculation, and statistical analyses were performed using GraphPad Prism software version 6.0 for Windows (GraphPad Software, San Diego, California, USA).

## RESULTS

**Figure 1:**
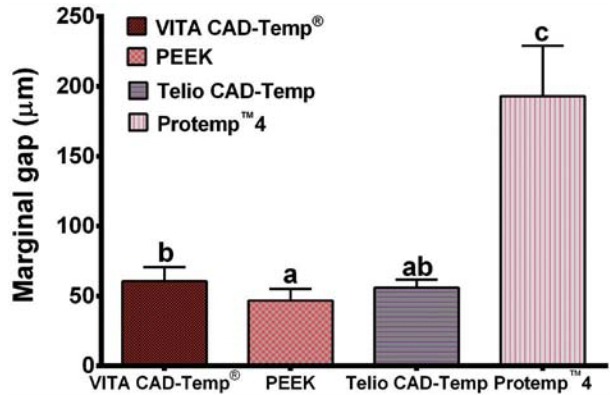
Bar graph showing the mean values and standard deviation (SD) of the marginal gap for each group of material. Means with the same letter are not significantly different. Means with different letters are significantly different

**Figure 2:**
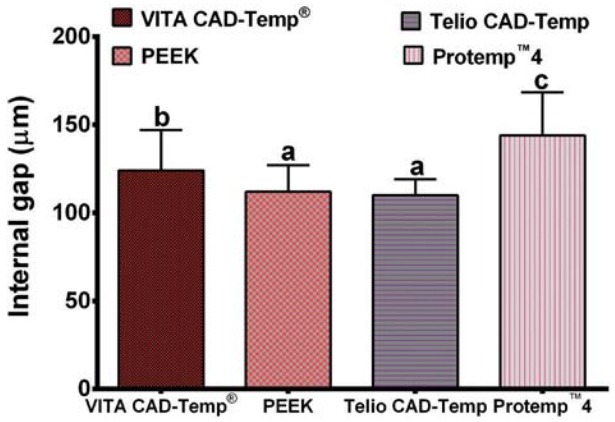
Bar graph showing the mean values and standard deviation (SD) of the internal gap for each group of material. Means with the same letter are not significantly different. Means with different letters are significantly different

**Figure 3:**
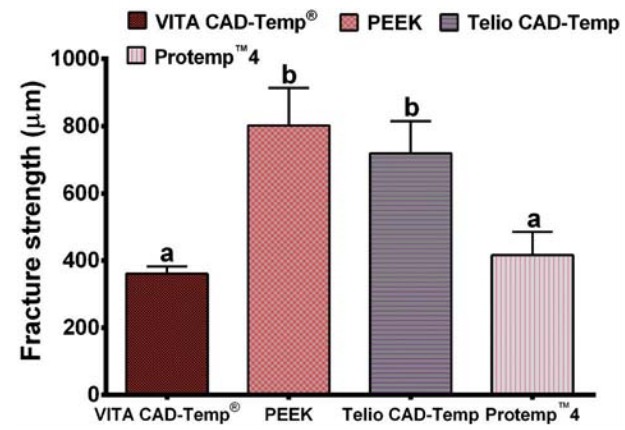
Bar graph showing the mean values and standard deviation (SD) of the fracture strength for each group of material. Means with the same letter are not significantly different. Means with different letters are significantly different

Mean values and standard deviations (M±SD) of the marginal gap, internal gap, and fracture strength for all groups are graphically shown in Figures 1 - 3.

The average marginal gap for each group was: VITA CAD-Temp^®^ 60.61±9.99 μm, PEEK 46.75±8.26 μm, Telio CAD-Temp 56.10±5.65 μm, and Protemp™4 193.07±35.96 μm. The statistical analysis of the results indicated that there was a statistically significant difference (p<0.001) between the groups. The average internal fit for each group was: VITA CAD-Temp^®^ group 124.94±22.96 μm, PEEK group 113.14±23.55 μm, Telio CAD-Temp group 110.95±11.64 μm, and Protemp^™^4 group 143.48±26.74 μm. The results of the internal fit between all groups showed a statistically significant difference (p<0.001). The average fracture strength of each group was: VITA CAD-Temp^®^ 361.01±21.61 N, PEEK 802.23±111.29 N, Telio CAD-Temp 719.24±95.17 N, and Protemp^™^4 416.40±69.14 N. The results of the fracture strength between all groups showed a statistically significant difference (p<0.001).

**Figure 4:**
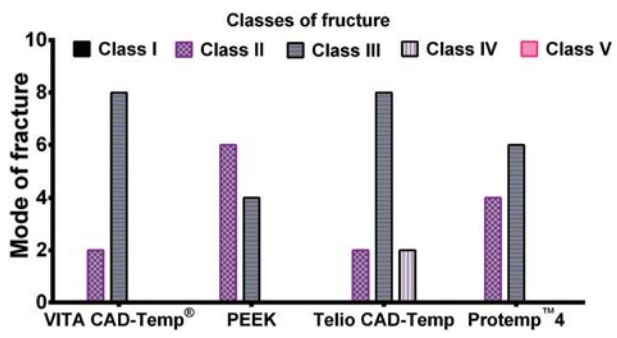
Bar graph showing the mode of failure for each material. For all groups the majority of the crowns had a Class III fracture, which is a fracture through the midline

One-way ANOVA test indicated that for the variables marginal gap, internal gap, and fracture strength, the p-value was lower than 0.05 for all the materials. Therefore, there was a statistically significant difference among the tested materials. However, the outcome of mode of fracture in the current study showed that there was no statistically significant difference between the groups (p>0.05) as shown in Figure 4.

## DISCUSSION

The purpose of this study was to investigate the difference in performance of provisional crowns that were fabricated either by a traditional direct technique or with the more sophisticated indirect CAD/CAM approach. The importance of precise provisional restorations is generally accepted in order for the definitive restoration to fit and function properly. In addition, there is certain evidence that the CAD/CAM provisional restorations may be superior to their direct counterparts^22^. The introduction of provisional CAD/CAM restorations promises a certainly easier method of fabrication for the clinician, but also offers potentially stronger provisional restorations. However, there may be an impetuous to use CAD/CAM provisional materials, these restorations comprise a more expensive alternative to conventional directly made provisional restorations. If the performance of these restorations is similar to conventionally fabricated provisional chairside restorations, then their use may be regarded as excessive.

A restoration, definitive or provisional, is regarded as successful when it exhibits a good marginal and internal fit and is strong enough to withstand the oral environment. Regarding the marginal adaptation, this is critical in the case of provisional restorations, since poor marginal fit can lead to inflammation of periodontal tissue, a situation that can postpone the fit of the definitive restorations. In literature there are different approaches to measure the marginal gap of restorations. Two common techniques to measure the marginal and internal gap are measurement of embedded and sectioned specimens, and measurement of the replica of the marginal and internal gap^2^ ^,^ ^10^. The replica technique is a non-invasive and valid technique to measure the adaptation of a restoration to the tooth structure^14^. In this study a replica technique that was used in a previous study was applied in order to evaluate the effectiveness of each material and method of provisional crown fabrication^25^.

The mean marginal gap of the CEREC provisional crowns reported in the current study ranged between 47-193 µm. These findings are far from the theoretically based requirements according to which the cementation film thickness should be between 25-40 µm^15^. However, in the literature, the marginal gaps reported for provisional crowns are well above the gap reported in this study^8^. In this study the marginal gap reported for Protemp^TM^4 was lower to that previously reported^6^ ^,^ ^24^. However, this could be due to the different methodology followed in the current study, since a replica technique was used to measure the marginal gap.

One of the problems reported for the directly made provisional restorations is the marginal discrepancies that occur due to polymerization shrinkage^6^ ^,^ ^17^. This problem is significantly greater with PMMA provisional materials and is comparatively less with bis-acryl composite resin materials^27^, but still poses a problem. CAD/CAM provisional materials do not face this issue as the restoration is milled from pre-polymerized blocks of the provisional material, in such a way that any degree of polymerization shrinkage has taken place during processing of the block. In this study, the CAD/CAM provisional crowns demonstrated lower marginal gaps compared with the direct counterpart. This result was consistent with a study by Yao, et al.^26^ (2014), in which it was found that the CAD/CAM provisional crowns had lower marginal gaps compared with direct provisional crowns^26^. However, when comparing the values reported, the marginal gap value in this study for Telio CAD-Temp and VITA CAD-Temp^®^ were lower. This could be due to the different methodology followed by Yao, et al.^26^ (2014), in which the crowns were cemented with glass ionomer cement^26^. Another interesting finding in the current study was that a statistically significant difference (p<0.001) was found between the CAD/CAM provisional materials, with the VITA CAD-Temp^®^ demonstrating the highest average marginal gap.

Regarding the internal fit of the restorations it was found that there was a statistically significant difference between the groups, with the Telio CAD-Temp group demonstrating the lower internal gap. A further interesting finding was that for the CAD/CAM groups, the greater average internal gap was found at the occlusal part of the restorations, while for the direct provisional material a more universal gap was found internally. This result was consistent with a previous study^25^ and may be due to the machining process of fabricating the crowns and the shape of the milling burs.

In order to measure the fracture strength of the provisional restorations, provisional cement was used to fit the crowns to simulate the clinical situation. The mean fracture strength reported in this study ranged from 361-802 N. The PEEK material demonstrated the highest fracture resistance, while VITA CAD-Temp^®^ demonstrated the lowest. A statistically significant difference was found between all groups (p<0.001). However, another study reported a non-significant result between provisional fabricated CAD/CAM fixed dental prosthesis with a directly made glass fiber material^19^.

The fracture strength exhibited by all tested materials occurred at loads above the maximum loads that can occur in the mouth in normal situations. It has been found that the produced force of a human is approximately 40 N during swallowing; 170–881 N during chewing nuts, and 39–788 N for corresponding mastication loads. In addition, this amount of force increases to optimum range from 200-540 N in the posterior (molar) region^5^ ^,^ ^18^ ^,^ ^24^. Therefore, these materials may not be able to withstand extreme occlusal forces in the oral environment.

Very little data exist in the literature regarding the fracture strength of these modern provisional materials. Ivoclar Vivadent in an *in vitro* study has reported that the fracture strength for Telio CAD-Temp and VITA CAD-Temp^®^ were approximately 1170 N and 605 N respectively^12^. However, it has been found that the fracture strength was about 300 N for anterior and 600 N for posterior crowns using PEEK material^11^. The results reported in this study differed from the above, which could be attributed to the different methodology. Regarding the fracture mode, the Kruskal-Wallis non-parametric analysis showed that there was no statistically significant difference between machinable and traditional provisional materials in the current study (p>0.05). The highest number of Type II fracture (less than half of crown fracture) was seen with PEEK and Protemp™4. One of the limitations of this study was that no fatigue loading was applied to the restorations. The average cyclic load during an individual life in an actual oral environment may exceed 10^7^ cycles, which can lead to up to 50% strength reduction, particularly in ceramic restorations because of fatigue^14^ ^,^ ^20^ ^,^ ^21^. The intention was to give a primary indication of the performance of the currently available CAD/CAM materials. However, in a study by Yao, et al.^26^ (2014), in which provisional materials were tested for their marginal adaptation prior to and after thermocycling, the results reported prior to thermocycling were similar to the results found in this study^26^. The next plan of the work in assessing these restorations is to subject them to more complex durability tests.

## CONCLUSION

Within the limitations of this study, the first part of the null hypothesis was partly accepted. The CAD/CAM provisional crowns demonstrated superior marginal fit compared with the direct provisional crowns. The mean internal gap was lower for the CAD/CAM groups; however, the internal gap was more uniform for the direct provisional crowns. Finally, the last part of the null hypothesis was rejected because not all CAD/CAM provisional crowns demonstrated superior fracture strength to that of the direct provisional material.

## References

[1] Alt V, Hannig M, Wöstmann B, Balkenhol M (2011). Fracture strength of temporary fixed partial dentures: CAD/CAM versus directly fabricated restorations. Dent Mater.

[2] Beschnidt SM, Strub JR (1999). Evaluation of the marginal accuracy of different all-ceramic crown systems after simulation in the artificial mouth. J Oral Rehabil.

[3] Burke FJ, Watts DC (1994). Fracture resistance of teeth restored with dentin-bonded crowns. Quintessence Int.

[4] Burns DR, Beck DA, Nelson SK (2003). Committee on Research in Fixed Prosthodontics of the Academy of Fixed Prosthodontics. A review of selected dental literature on contemporary provisional fixed prosthodontic treatment: report of the Committee on Research in Fixed Prosthodontics of the Academy of Fixed Prosthodontics. J Prosthet Dent.

[5] Las Casas EB, Almeida AF, Cimini CA, Gomes PT, Cornacchia TP, Saffar JM (2007). Determination of tangential and normal components of oral forces. J Appl Oral Sci.

[6] Ehrenberg D, Weiner GI, Weiner S (2006). Long-term effects of storage and thermal cycling on the marginal adaptation of provisional resin crowns: a pilot study. J Prosthet Dent.

[7] Fasbinder DJ (2006). Clinical performance of chairside CAD/CAM restorations. J Am Dent Assoc.

[8] Givens EJ, Neiva G, Yaman P, Dennison JB (2008). Marginal adaptation and color stability of four provisional materials. J Prosthodont.

[9] Hamza TA, Rosenstiel SF, Elhosary MM, Ibraheem RM (2004). The effect of fiber reinforcement on the fracture toughness and flexural strength of provisional restorative resins. J Prosthet Dent.

[10] Holmes JR, Bayne SC, Holland GA, Sulik WD (1989). Considerations in measurement of marginal fit. J Prosthet Dent.

[11] Invibio Biomaterial Solutions (2011). New material options for innovations in restorative and prosthetic dentistry.

[12] Ivoclar-Vivadent (2010). Telio CAD scientific documentation.

[13] Jedynakiewicz NM, Martin N (2001). CEREC: science, research, and clinical application. Compend Contin Educ Dent.

[14] Jung YG, Peterson IM, Kim DK, Lawn BR (2000). Lifetime-limiting strength degradation from contact fatigue in dental ceramics. J Dent Res.

[15] McLean JW, von Fraunhofer JA (1971). The estimation of cement film thickness by an in vivo technique. Br Dent J.

[16] Mormann WH (2006). The evolution of the CEREC system. J Am Dent Assoc.

[17] Nejatidanesh F, Lotfi HR, Savabi O (2006). Marginal accuracy of interim restorations fabricated from four interim autopolymerizing resins. J Prosthet Dent.

[18] Pallis K, Griggs JA, Woody RD, Guillen GE, Miller AW (2004). Fracture resistance of three all-ceramic restorative systems for posterior applications. J Prosthet Dent.

[19] Peñate L, Basilio J, Roig M, Mercadé M (2015). Comparative study of interim materials for direct fixed dental prostheses and their fabrication with CAD/CAM technique. J Prosthet Dent.

[20] Peterson IM, Wuttiphan S, Lawn BR, Chyung K (1998). Role of microstructure on contact damage and strength degradation of micaceous glass-ceramics. Dent Mater.

[21] Powers JM, Sakaguchi RL (2012). Craig’s restorative dental materials.

[22] Rayyan MM, Aboushelib M, Sayed NM, Ibrahim A, Jimbo R (2015). Comparison of interim restorations fabricated by CAD/CAM with those fabricated manually. J Prosthet Dent.

[23] Strassler HE, Lowe RA (2011). Chairside resin-based provisional restorative materials for fixed prosthodontics. Compend Contin Educ Dent.

[24] Strub JR, Beschnidt SM (1998). Fracture strength of 5 different all-ceramic crown systems. Int J Prosthodont.

[25] Tsitrou EA, Northeast SE, van Noort R (2007). Evaluation of the marginal fit of three margin designs of resin composite crowns using CAD/CAM. J Dent.

[26] Yao J, Li J, Wang Y, Huang H (2014). Comparison of the flexural strength and marginal accuracy of traditional and CAD/CAM interim materials before and after thermal cycling. J Prosthet Dent.

[27] Young HM, Smith CT, Morton D (2001). Comparative in vitro evaluation of two provisional restorative materials. J Prosthet Dent.

